# Supplementary material for: Rewired m6A epitranscriptomic networks link mutant p53 to neoplastic transformation
Source: Nat Commun. 2023 Mar 27;14:1694. doi: 10.1038/s41467-023-37398-9 (PMC10042811; doi:10.1038/s41467-023-37398-9)
Supplement: Supplementary file 1 — Supplementary Information [file 41467_2023_37398_MOESM1_ESM.pdf]

Supplementary Information

Rewired m^{6}A epitranscriptomic networks link mutant p53 to neoplastic transformation

An Xu, Mo Liu, Mo-Fan Huang, Yang Zhang, Ruifeng Hu, Julian A. Gingold, Ying Liu, Dandan Zhu, Chian-Shiu Chien, Wei-Chen Wang, Zian Liao, Fei Yuan, Chih-Wei Hsu, Jian Tu, Yao Yu, Taylor Rosen, Feng Xiong, Peilin Jia, Yi-Ping Yang, Danielle A. Bazer, Ya-Wen Chen, Wenbo Li, Chad D. Huff, Jay-Jiguang Zhu, Francesca Aguilo, Shih-Hwa Chiou, Nathan C Boles, Chien-Chen Lai, Mien-Chie Hung, Zhongming Zhao, Eric L Van Nostrand, Ruiying Zhao & Dung-Fang Lee

Supplementary Figure 1

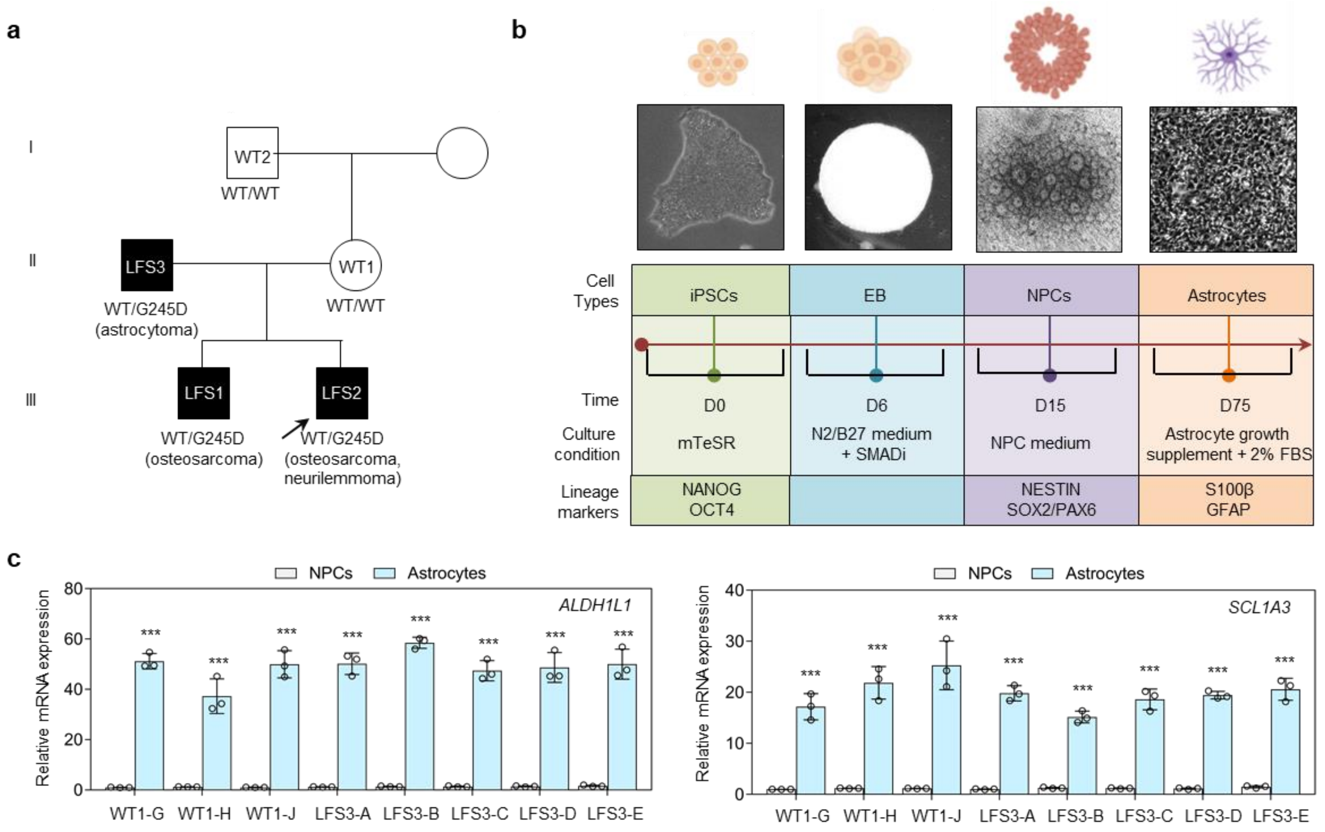

Supplementary Fig. 1. Differentiation of LFS iPSCs into NPCs and astrocytes. a. LFS family tree. c.734G>A mutation in the *TP53* gene causing the G245D mutation is found in LFS patients. Arrow, proband. **b.** Schematic diagram of astrocyte differentiation. The differentiation of iPSCs into astrocytes is driven by timed maintenance in the defined culture medium supplemented with special growth factors and inhibitors and combined with a transition from two- to three-dimensional culture. Developmental and maturation lineage markers are utilized to monitor the differentiation progress at each differentiation stage (iPSCs, EB, NPCs, and astrocytes). The illustration is created with BioRender.com. **c.** RT-qPCR for expression of astrocyte marker genes (ALDH1L1 and SLC1A3) in the WT and LFS astrocytes at D75 (n=3 biologically independent samples). The result is representative of three independent experiments. The data are presented as the mean ± SEM; two-way ANOVA with Bonferroni's multiple comparison test. ***$p < 0.001$. Source data and exact p values are provided in the Source Data file.

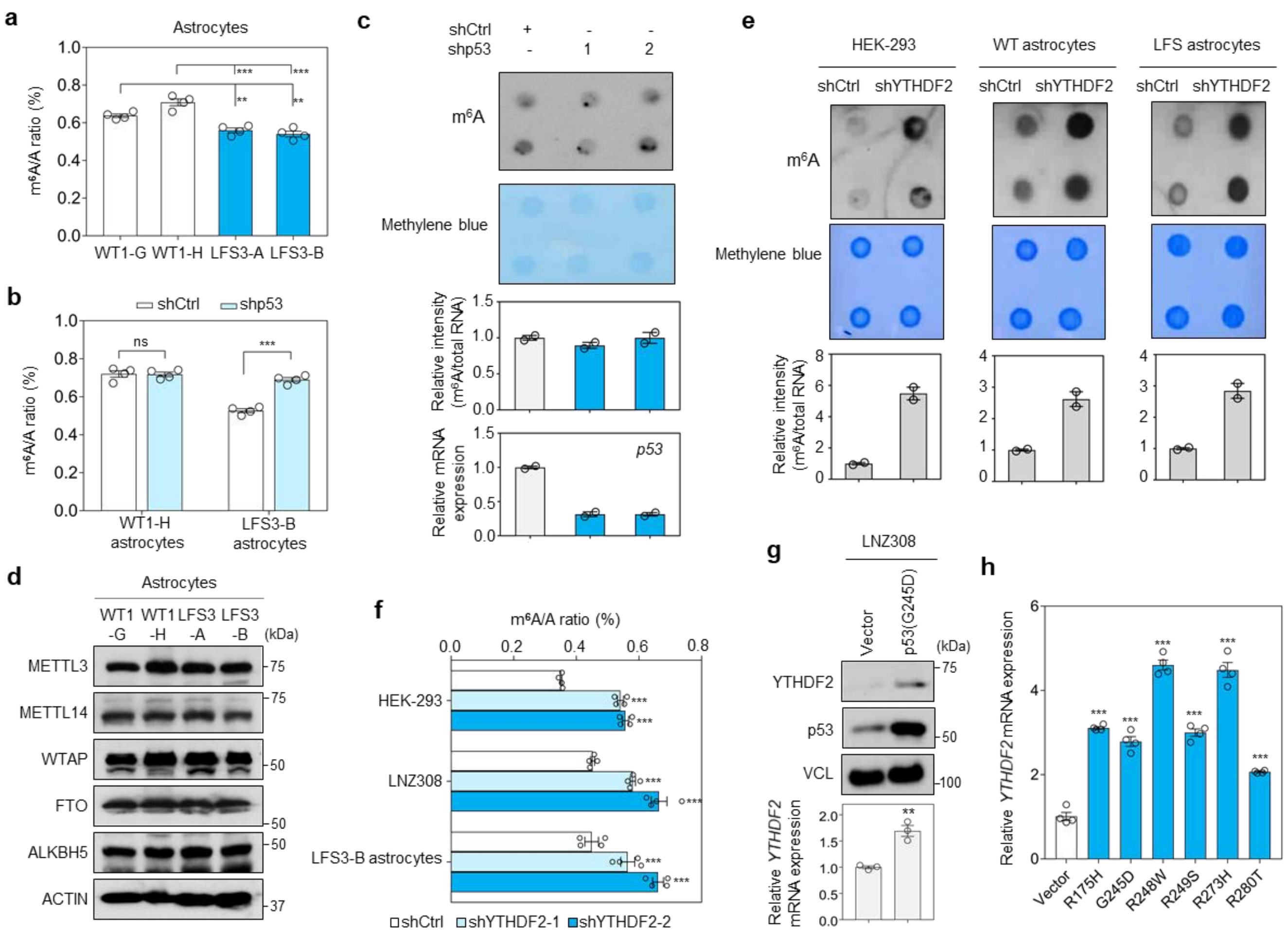

Supplementary Fig. 2. YTHDF2-mediated m^{6}A degradation is modulated by mutant p53. a. Mass spectrometry analysis of m^{6}A/A ratio in WT and LFS astrocytes (n=4 biologically independent samples). **b.** Mass spectrometry analysis of m^{6}A/A ratio in p53-depleted WT and LFS astrocytes (n=4 biologically independent samples). **c.** m^{6}A methylation dot blotting indicates p53 knockdown does not influence m^{6}A methylation in WT astrocytes. Dot blot analysis is performed to identify polyadenylated mRNA isolated from shCtrl and shp53 transduced WT astrocytes and immunoblotted with anti-m^{6}A antibodies (top blot). Methylene blue staining of total mRNA is used as a loading control (bottom blot). Dot density is measured by ImageJ. The blotting images represent the results of at least three independent experiments, while the bar charts depict technical replicates within a single experiment. **d.** Immunoblotting indicates no significant differences of METTL3, METTL14, WTAP, FTO, and ALKBH5 expression between WT and LFS astrocytes. **e.** m^{6}A methylation dot blot confirms the role of YTHDF2 in degrading m^{6}A-marked transcripts. Dot blots identify polyadenylated mRNA isolated from shCtrl and shYTHDF2 transduced HEK293T cells as well as WT and LFS astrocytes following immunoblotting with anti-m^{6}A antibodies (top

blot). Methylene blue staining of total mRNA is used as a loading control (bottom blot). Dot density is measured by ImageJ. The blotting images represent the results of at least three independent experiments, while the bar charts depict technical replicates within a single experiment. **f.** Mass spectrometry analysis of m^6A/A ratio in YTHDF2-depleted HEK-293, LNZ308, and LFS astrocytes (n=4 biologically independent samples). **g**. Immunoblotting indicates p53(R175H) upregulates YTHDF2 protein in LNZ308 cells (n=3 biologically independent samples). **h**. Expression of distinct mutant p53s (R175H, G245D, R248W, R273H, and R280T) increases *YTHDF2* mRNA in WT astrocytes (n=4 biologically independent samples). The results are representative of at least three independent experiments (**c**-**e**, **g**). The data are presented as the mean ± SEM; one-way ANOVA with Tukey's multiple comparison test (**a**, **h)**; two-way ANOVA with Bonferroni's multiple comparison test (**b**, **f)**; unpaired two-tailed Student's *t* test (**g**). $^{**}p < 0.01$, $^{***}p < 0.001$, ns, not significant. Source data and exact p values are provided in the Source Data file.

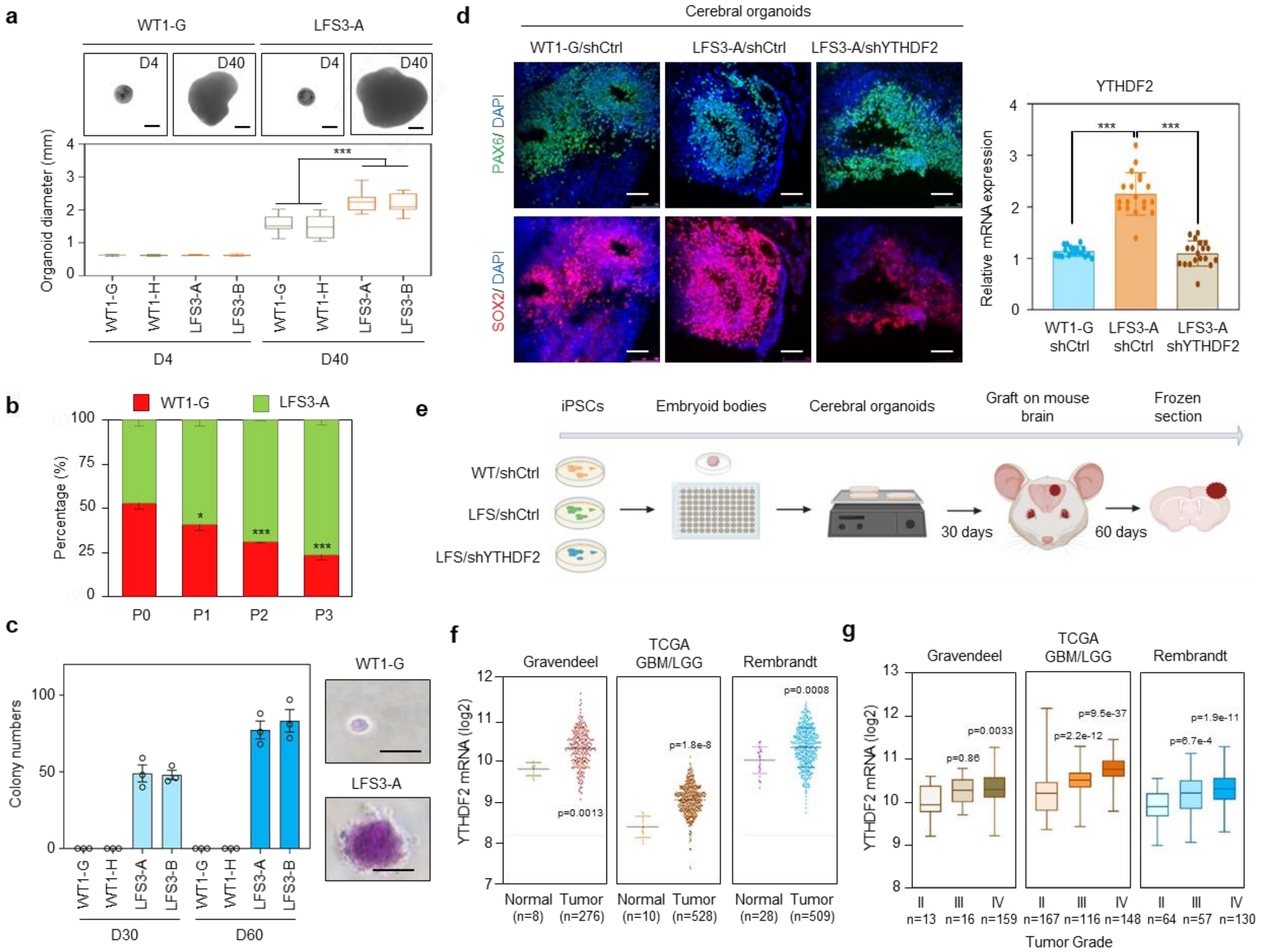

Supplementary Fig. 3. LFS astrocytes and cerebral organoids show oncogenic properties. a. Cerebral organoid culture demonstrates the markedly increased size of LFS organoids. Representative WT and LFS organoids (n=19 biologically independent samples) at day 4 (D4) and day 40 (D40) are shown in the upper panels. The average diameters of WT and LFS organoids are quantified in the lower panels. Scale bar, 500μm. Box edges delineate lower and upper quartiles, the center line represents the median, and whiskers extend to 1.5 times the interquartile range. **b.** Fluorescence-based competition assay indicates higher proliferation ability of LFS astrocytes compared to WT astrocytes at day 120 (n=3 biologically independent samples). **c.** AIG assay for *in vitro* tumorigenicity demonstrates that LFS but not WT astrocytes are capable of growth in soft agar. Colonies are counted and measured at day 30 (D30) and day 60 (D60) (n=3 biologically independent samples). LFS astrocytes demonstrate increased total colony numbers during culture. Scale bar, 50μm. **d.** Immunofluorescence staining of cerebral organoids. One-month-old cerebral organoids of WT/shCtrl, LFS/shCtrl, and LFS/shYTHDF2 present similar

patterns of forebrain identities based on $SOX2^{+}$ and $PAX6^{+}$ staining (n=19 biologically independent samples). Scale bar, 50µm. **e.** Illustration of the experimental procedure for cerebral organoid generation and transplantation on mouse brains. The illustration is created with BioRender.com. **f-g.** Dot and box plots of *YTHDF2* mRNA expression profiles (log2) of glioma specimens in Gravendeel, The Cancer Genome Atlas (TCGA) GBM/LGG, and Rembrandt datasets show high YTHDF2 expression in tumor specimens compared to normal tissues (**f**) and increasing YTHDF2 expression in the higher grade of gliomas (**g**). Box edges delineate lower and upper quartiles, the center line represents the median, and whiskers extend to 1.5 times the interquartile range. The data are presented as the mean ± SEM; one-way ANOVA with Tukey's multiple comparison test (**a**, **c**, **d**); unpaired two-tailed Student's *t* test (**b**, **f**, **g**). *p < 0.05, ***p < 0.001, Source data and exact p values are provided in the Source Data file.

Supplementary Figure 4

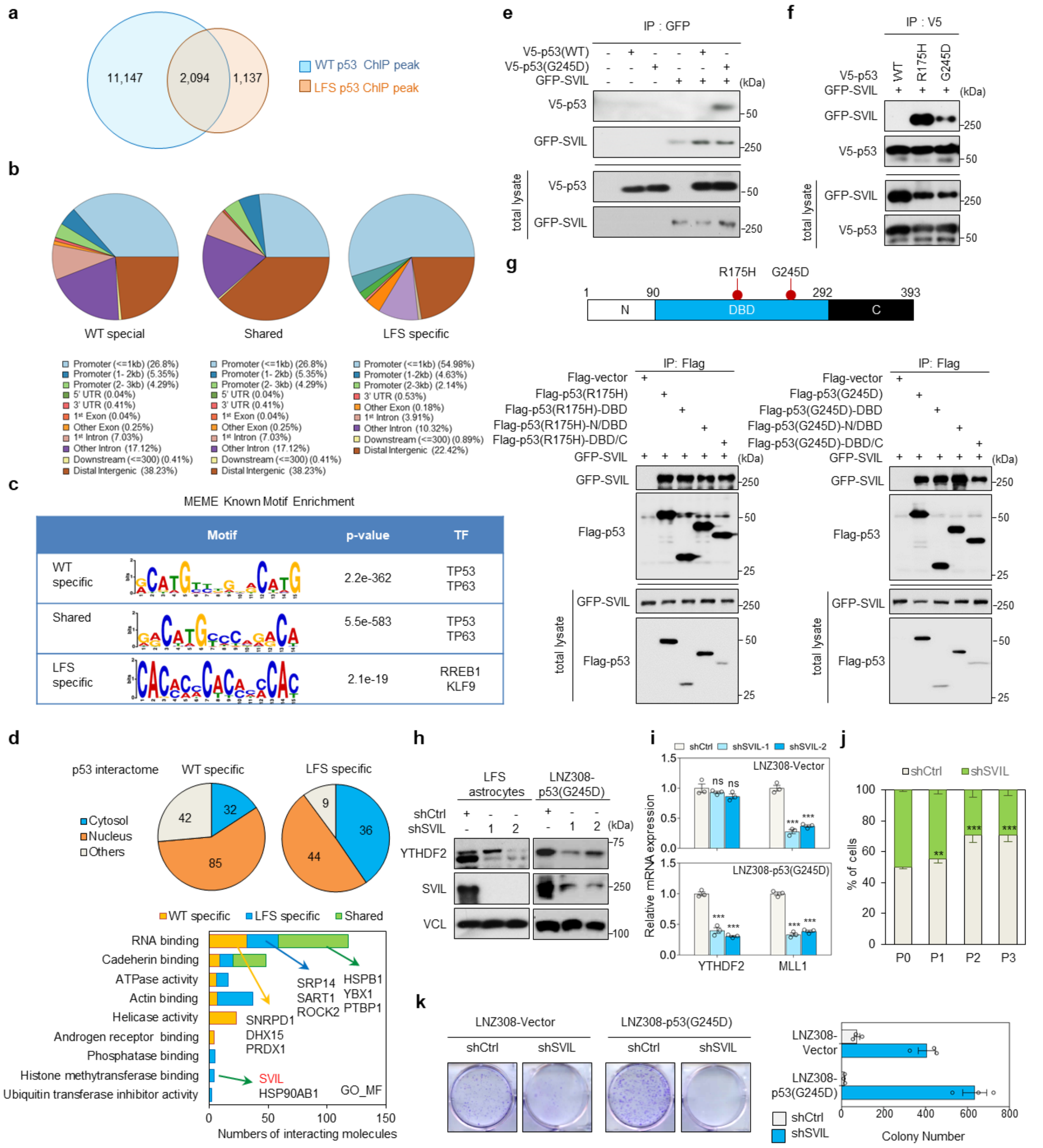

Supplementary Fig. 4. Genome-wide binding of p53 and mutant p53 in WT and LFS astrocytes a. Venn diagram shows the overlap between p53 and mutant p53 genome binding peaks in WT and LFS astrocytes, respectively. **b.** Pie charts report the genomic distribution annotation of p53-specific, mutant

p53-specific, and shared genome binding peaks in WT and LFS astrocytes. **c.** MEME motif analyses discover the top-scoring binding motif for p53-specific, mutant p53-specific, and shared genome binding peaks in WT and LFS astrocytes. **d.** Gene Ontology Molecular Function (GO_MF) analysis of p53 and mutant p53 interacting proteins. SVIL is a mutant p53-specific interacting protein involved in histone methyltransferase binding. **e.** p53(G245D) but not p53 interacts with SVIL exogenously. Co-IP is performed by pulling down GFP-tagged SVIL in lysates of HEK-293T cells co-transfected with GFP-tagged SVIL and V5-tagged p53(G245D) or p53. **f.** Both p53(R175H) and p53(G245D) physically associate with SVIL. Co-IP is performed by pulling down V5-tagged p53(R175H) or p53(G245D) in lysates of HEK-293T cells cotransfected with GFP-tagged SVIL and p53(R175H) or p53(G245D). **g.** The DNA binding domain (DBD) of mutant p53 interacts with SVIL. Co-IP maps the essential domains (N-terminal (1-90 aa), DBD (91-292 aa), and C-terminal (293-393 aa)) of mutant p53 (p53(R175H) and p53(G245d)) interacting with SVIL. Co-IP is performed by pulling down Flag-tagged p53 domains in lysates of HEK-293T cells co-transfected with GFP-tagged SVIL and Flag-tagged p53, p53(DBD), p53(N/DBD), or p53(DBD/C). **h.** Immunoblotting indicates that SVIL depletion leads to downregulated YTHDF2 protein expression in both LFS astrocytes and LNZ308-p53(G245D) cells. **i.** RT-qPCR analysis shows decreased *YTHDF2* mRNA expression upon SVIL knockdown in LNZ308-p53(G245D) but not LNZ308-Vector cells (n=3 biologically independent samples). **j.** Fluorescence-based competition assay indicates reduced growth of SVIL-depleted LFS astrocytes compared with co-cultured LFS astrocytes. LFS astrocytes are transduced with lentiviruses carrying control shRNA (shCtrl) or SVIL shRNA (shSVIL with GFP marker). The mixed LFS/shCtrl and LFS/shSVIL astrocytes are co-cultured and cell populations examined by flow cytometry (n=5 biologically independent samples). **k.** SVIL knockdown more significantly inhibits colony-forming ability in LNZ308-p53(G245D) cells than LNZ308-Vector cells (n=3 biologically independent samples). Colony numbers are calculated by ImageJ. The results are representative of at least three independent experiments (**e**-**h**). The data are presented as the mean ± SEM; two-way ANOVA with Bonferroni's multiple comparison test (**I**, **k**); unpaired two-tailed Student's *t* test (**j**). $^{**}p < 0.01$, $^{***}p < 0.001$, ns, not significant. Source data and exact p values are provided in the Source Data file.

Supplementary Figure 5

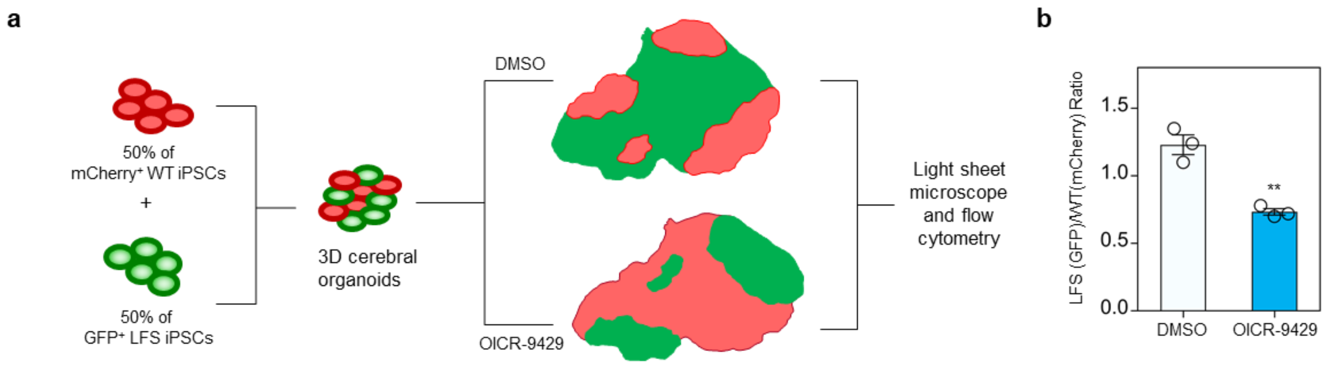

Supplementary Fig. 5. Inhibition of MLL1 function selectively impairs LFS cerebral organoid proliferation. a. Illustration of the experimental design for the 3D competition assay using WT/LFS cerebral organoids. **b.** Calculations of WT (mCherry$^+$) and LFS (GFP$^+$) cell percentages in WT/LFS cerebral organoids by light sheet fluorescence microscopy (n=3 biologically independent samples). The data are presented as the mean ± SEM; unpaired two-tailed Student's *t* test. **p < 0.01. Source data and exact p values are provided in the Source Data file.

Supplementary Figure 6

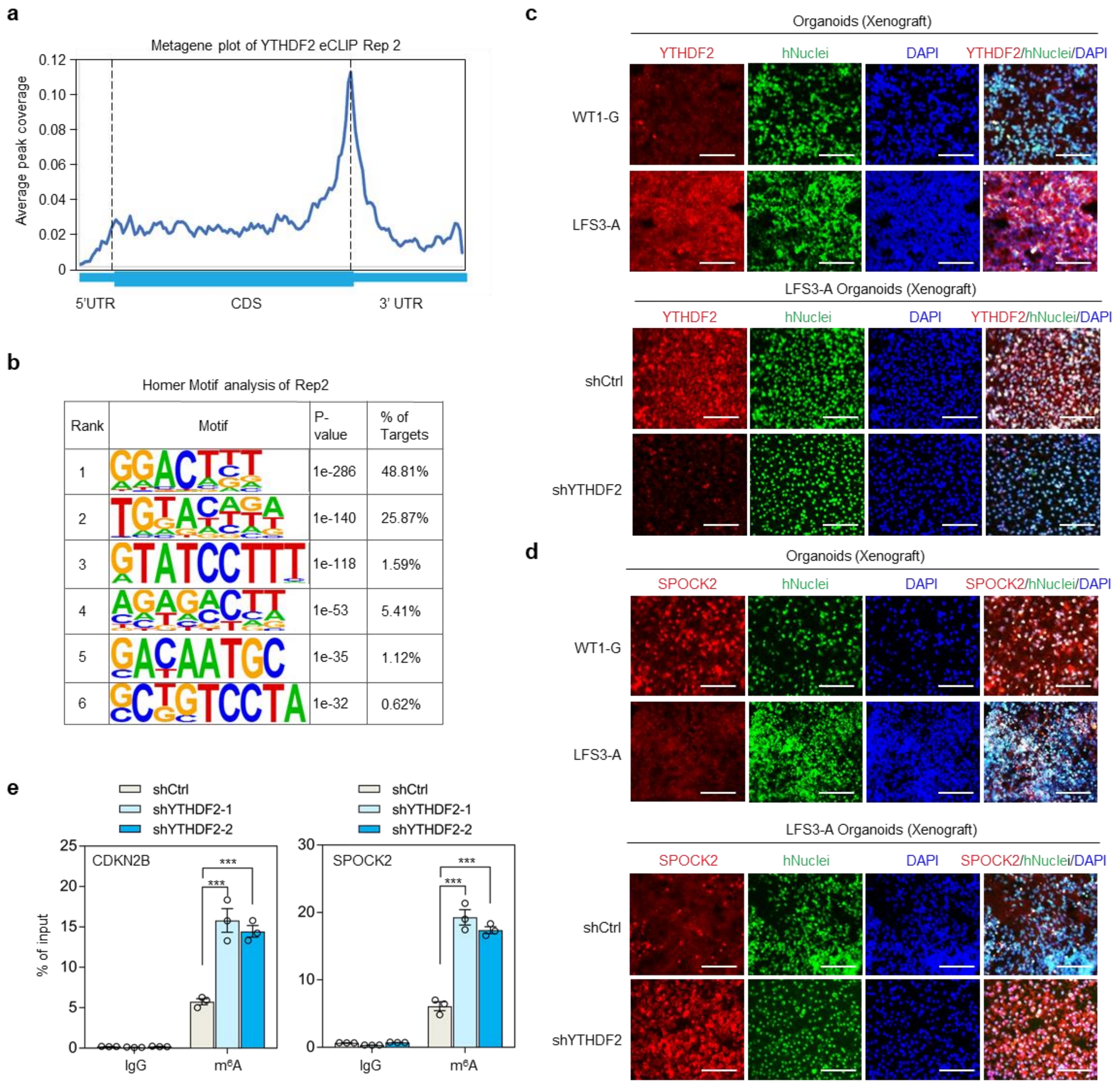

Supplementary Fig. 6. YTHDF2 eCLIP-seq and upregulation of *CDKN2B* and *SPOCK2* transcripts upon YTHDF2 knockdown. a. Metagene plot of YTHDF2 eCLIP-seq indicates enrichment of YTHDF2-interacting mRNA peaks in the 3'UTR centered around stop codons in a replicate experiment (Rep2). **b.** Motif analysis demonstrates YTHDF2 binding motifs in a replicate experiment (Rep2). **c.** Immunostaining confirms higher YTHDF2 in engrafted LFS cerebral organoids (upper panel) and decreased YTHDF2 in YTHDF2-depleted engrafted LFS cerebral organoids (lower panel). Scale bar, 100μm. **d.** Immunostaining demonstrates lower SPOCK2 in engrafted LFS cerebral organoids (upper panel) and increased SPOCK2

in YTHDF2-depleted engrafted LFS cerebral organoids (lower panel). Scale bar, 100µm. **e.** m^6A MeRIP-PCR indicates that depletion of YTHDF2 leads to an increase of m^6A-modified *CDKN2B* and *SPOCK2* mRNAs (n=3 biologically independent samples). The results are representative of at least three independent experiments (**c**-**d)**. The data are presented as the mean ± SEM; two-way ANOVA with Bonferroni's multiple comparison test. ***p < 0.001. Source data and exact p values are provided in the Source Data file.

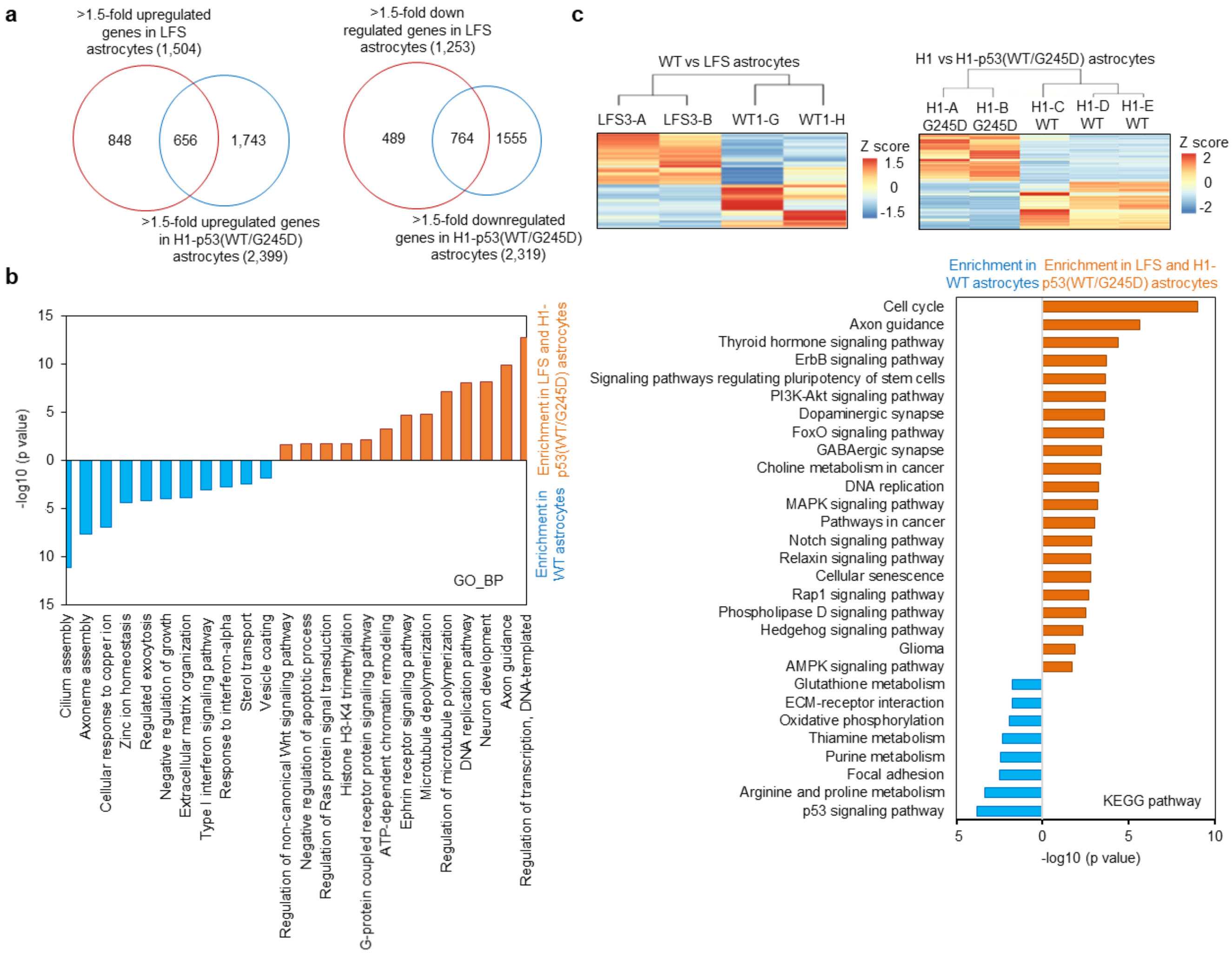

Supplementary Fig. 7. LFS astrocytes acquire oncogenic properties. a. Venn diagram indicates the number of significantly (fold change >1.5; p<0.01) differentially upregulated or downregulated genes between LFS or H1-p53(WT/G245D) astrocytes compared with WT and H1-WT astrocyte controls and the overlap between each set of genes. **b.** GO_BP analysis of enriched genes in LFS and H1-p53(WT/G245D) astrocytes (orange columns) or WT and H1-WT astrocytes (blue columns). **c.** KEGG pathway analysis of enriched genes identified in either p53 wild-type or p53-mutant astrocytes. Upper panel, hierarchical clustering of transcriptome Z-score computed for all genes that are differentially expressed (more than two-fold, p-value <0.01) between all pairwise comparisons of WT and LFS astrocytes or H1-WT and H1-p53(WT/G245D) astrocytes. Lower panel, Enrichr analysis reveals KEGG pathways enriched in WT and H1-WT astrocytes (blue columns) or LFS and H1-p53(WT/G245D) astrocytes (orange columns).

Supplementary Figure 8

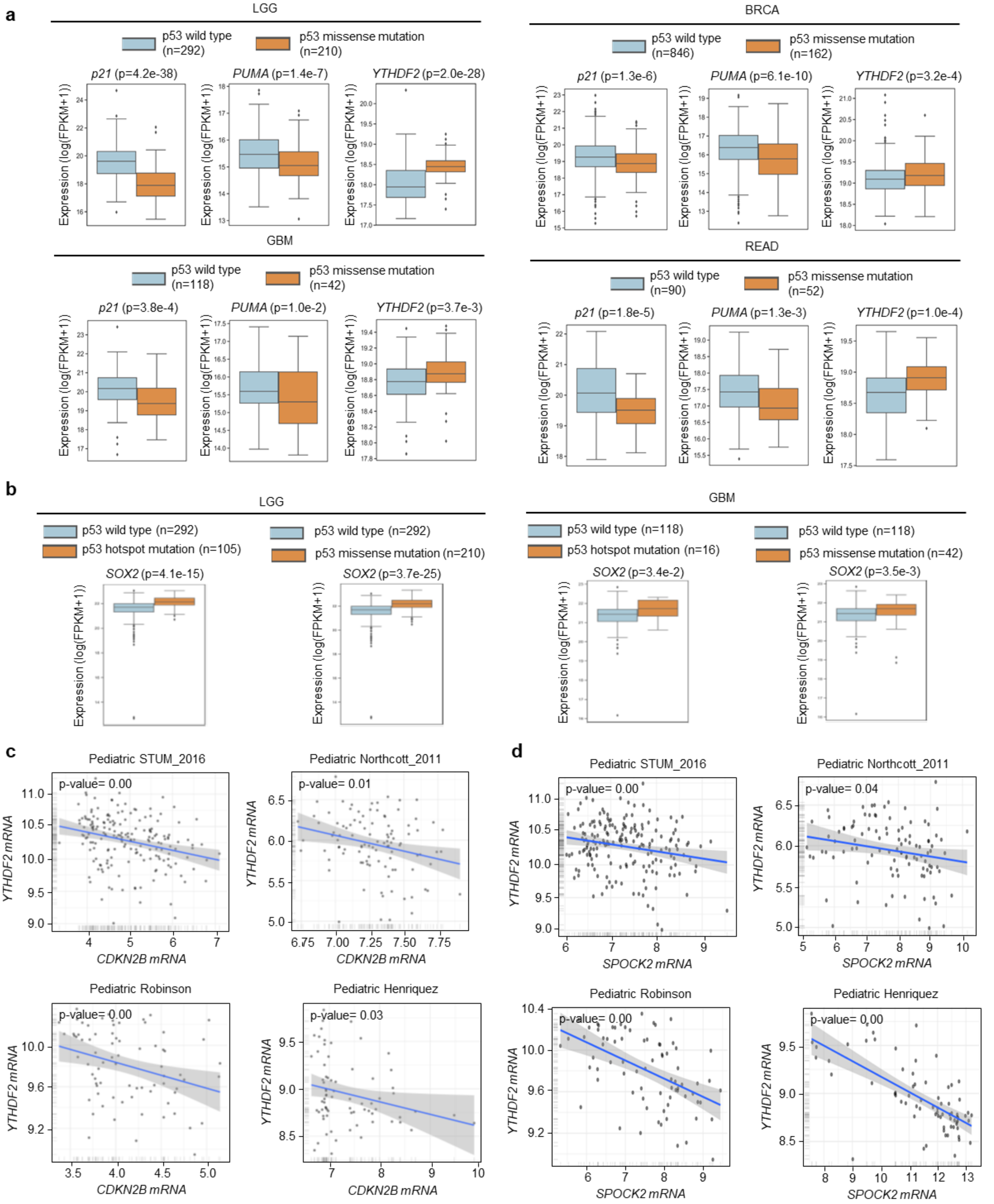

Supplementary Fig. 8. Clinical correlation studies in human tumor specimens. a. Clinical correlation between mutant p53 and YTHDF2 in LGG, GBM, BRCA, and READ tumors. Box plots of TCGA RNA expression profiles (log2) in TCGA tumors with p53 wild-type or p53 missense mutation genotypes in LGG, GBM, BRCA, and READ specimens. Correlation analysis of *p21*, *PUMA*, and *YTHDF2* mRNA levels in p53 wild-type or p53 missense mutations in LGG, GBM, BRCA, and READ tumors demonstrates elevated Y*THDF2* mRNA expression but decreased *p21* and *PUMA* mRNA expression in tumors with p53 missense mutations. Box edges delineate lower and upper quartiles, the center line represents the median, and whiskers extend to 1.5 times the interquartile range. Two-sided Mann Whitney Wilcoxon test is performed to compute significance. **b.** Clinical correlation analysis of *SOX2* mRNA levels in LGG and GBM tumors with p53 wild-type or p53 missense mutation genotypes demonstrate elevated *SOX2* mRNA expression in tumors with p53 missense mutations. Box edges delineate lower and upper quartiles, the center line represents the median, and whiskers extend to 1.5 times the interquartile range. Two-sided Mann Whitney Wilcoxon test is performed to compute significance. **c-d.** High YTHDF2 expression is correlated with low CDKN2B (**c**) and SPOCK2 (**d**) expression in pediatric brain tumor datasets (Pediatric STUM_2016, Pediatric Northcott_2011, Pediatric Robison, and Pediatric Henriquez). Pearson's product-moment correlation is performed to compute significance. The error bands indicate the range of the upper and lower bound of the confidence interval for the predicted values.
