# Supplementary material for: Rewired m6A epitranscriptomic networks link mutant p53 to neoplastic transformation
Source: Nat Commun. 2023 Mar 27;14:1694. doi: 10.1038/s41467-023-37398-9 (PMC10042811; doi:10.1038/s41467-023-37398-9)
Supplement: Supplementary file 3 — Description of Additional Supplementary Files [file 41467_2023_37398_MOESM3_ESM.pdf]

Description of Additional Supplementary Files

File Name: Supplementary Data 1
Description: Peak annotation of p53 ChIP targets in WT and LFS astrocytes

File Name: Supplementary Data 2
Description: IP-MS identifies p53 and mutant p53 interacting proteins

File Name: Supplementary Data 3
Description: IP-MS identifies SVIL interacting proteins

File Name: Supplementary Data 4
Description: Peak annotation of m6A MeRIP targets in LFS astrocytes

File Name: Supplementary Data 5
Description: Peak annotation of YTHDF2 eCLIP targets in LFS astrocytes (Repcate #1 and #2)

File Name: Supplementary Data 6
Description: shRNA sequences and primers used for RT-qPCR, and ChIP-PCR
