# Supplementary material for: Rewired m6A epitranscriptomic networks link mutant p53 to neoplastic transformation
Source: Nat Commun. 2023 Mar 27;14:1694. doi: 10.1038/s41467-023-37398-9 (PMC10042811; doi:10.1038/s41467-023-37398-9)
Supplement: Supplementary file 10 — Reporting Summary [file 41467_2023_37398_MOESM10_ESM.pdf]

nature portfolio

Corresponding author(s): Dung-Fang Lee and Ruiying Zhao

Last updated by author(s): Feb 26, 2023

Reporting Summary

Nature Portfolio wishes to improve the reproducibility of the work that we publish. This form provides structure for consistency and transparency in reporting. For further information on Nature Portfolio policies, see our Editorial Policies and the Editorial Policy Checklist.

Statistics

For all statistical analyses, confirm that the following items are present in the figure legend, table legend, main text, or Methods section.

n/a	Confirmed	
☐	☒	The exact sample size (n) for each experimental group/condition, given as a discrete number and unit of measurement
☐	☒	A statement on whether measurements were taken from distinct samples or whether the same sample was measured repeatedly
☐	☒	The statistical test(s) used AND whether they are one- or two-sided<br>*Only common tests should be described solely by name; describe more complex techniques in the Methods section.*
☒	☐	A description of all covariates tested
☐	☒	A description of any assumptions or corrections, such as tests of normality and adjustment for multiple comparisons
☐	☒	A full description of the statistical parameters including central tendency (e.g. means) or other basic estimates (e.g. regression coefficient) AND variation (e.g. standard deviation) or associated estimates of uncertainty (e.g. confidence intervals)
☐	☒	For null hypothesis testing, the test statistic (e.g. F, t, r) with confidence intervals, effect sizes, degrees of freedom and P value noted<br>*Give P values as exact values whenever suitable.*
☒	☐	For Bayesian analysis, information on the choice of priors and Markov chain Monte Carlo settings
☒	☐	For hierarchical and complex designs, identification of the appropriate level for tests and full reporting of outcomes
☐	☒	Estimates of effect sizes (e.g. Cohen's d, Pearson's r), indicating how they were calculated

Our web collection on statistics for biologists contains articles on many of the points above.

Software and code

Policy information about availability of computer code

Data collection	Histology data for H&E was performed by Taipei Veterans General Hospital. RT-qPCR data was acquired using CFX96 real-time PCR system, Bio-Rad. Western blot data was acquired using films. RNA-seq, ChIP-seq, MeRIP-seq, and eCLIP-seq data have been deposited on GEO under the accession number provided in the methods section.
Data analysis	Image scan: EPSON V550 Photo<br>Western blot image processing: Adobe Photoshop CS5<br>RNA-seq analysis: Galaxy Community Hub (https://galaxyproject.org/) and Cutadapt 4.2<br>ChIP-seq analysis: Trim Galore! (version 0.6.5), Bowtie2 v2.5.1, MACS2 2.2.7.1, SAMtools 1.12, deepTools 3.5.0, and HOMER<br>Flow cytometry data collection: BD FACSDiva v.6.1.3<br>Flow cytometry data analysis: FlowJo v8<br>Genomic track view: IGV 2.16.0<br>The signal density analysis: ImageJ (version 1.53t 24)<br>Statistical analysis: GraphPad Prism 8.0 and Microsoft Excel (Office 2000)<br>The scripts for eCLIP analysis: https://github.com/VanNostrandLab/eclip<br>The scripts for TCGA analysis : https://github.com/huruifeng/m6A_p53_TCGA

For manuscripts utilizing custom algorithms or software that are central to the research but not yet described in published literature, software must be made available to editors and reviewers. We strongly encourage code deposition in a community repository (e.g. GitHub). See the Nature Portfolio guidelines for submitting code & software for further information.

Data

Policy information about availability of data

All manuscripts must include a data availability statement. This statement should provide the following information, where applicable:

- Accession codes, unique identifiers, or web links for publicly available datasets
- A description of any restrictions on data availability
- For clinical datasets or third party data, please ensure that the statement adheres to our policy

The raw and processed RNA-seq, ChIP-seq, MeRIP-seq, and eCLIP-seq data are deposited on National Center for Biotechnology Information Gene Expression Omnibus (NCBI-GEO) (GSE163088; https://www.ncbi.nlm.nih.gov/geo/query/acc.cgi?acc=GSE163088). Databases used in this study include the TRC library database (https://www.broadinstitute.org/rnai-consortium/rnai-consortium-shrna-library), The Cancer Genome Atlas (TCGA) (https://tcga-data.nci.nih.gov/tcga/), GlioVis Data Visualization Tools for Brain Tumor Datasets (http://gliovis.bioinfo.cnio.es/), and Enrichr (https://maayanlab.cloud/Enrichr/). The remaining data are available within the Article and Supplementary Information. Source data are provided in this paper.

Human research participants

Policy information about studies involving human research participants and Sex and Gender in Research.

Reporting on sex and gender	The study included all glioma patients with both genders, regardless of age range or molecular subtypes.
Population characteristics	The study included all glioma patients with both genders, regardless of age range or molecular subtypes. Age, gender, genotypic background, and therapeutic history were not treated as covariates in this study.
Recruitment	Glioma pecimens were pre-collected in Taipei Veterans General Hospital. Written informed consent was obtained from all patients. This study was not involved any patient recruitment.
Ethics oversight	The Institutional Ethics Committee/Institutional Review Board of Taipei Veterans General Hospital

Note that full information on the approval of the study protocol must also be provided in the manuscript.

Field-specific reporting

Please select the one below that is the best fit for your research. If you are not sure, read the appropriate sections before making your selection.

☒ Life sciences ☐ Behavioural & social sciences ☐ Ecological, evolutionary & environmental sciences

For a reference copy of the document with all sections, see nature.com/documents/nr-reporting-summary-flat.pdf

Life sciences study design

All studies must disclose on these points even when the disclosure is negative.

Sample size	Sample sizes for in vitro and in vivo experiments were determined on the basis of prior knowledge of variation, including our previous studies (Lee et al. Cell. 2015 PMID: 25860607; Kim et al. Proc Natl Acad Sci U S A. 2018. PMID: 30385632; Tu et al. Proc Natl Acad Sci U S A. 2022. PMID: 35412907). No statistical method was used to predetermine sample size as sample size selection with the above-published methods is sufficient to detect meaningful biological differences with good reproducibility.
Data exclusions	No data was excluded from the studies.
Replication	Biological replicates of each experiment were stated under each figure legend and all attempts were successful. Moreover, findings were repeatedly reproduced throughout the study: RNA-seq with RT-qPCR in multiple cell lines; protein levels with immunoblotting and immunostaining; and cell response assays with different cell line models.
Randomization	For animal experiments, age, and sex-matched mice were randomized into control and experimental groups. For in vitro experiments, cells were randomly allocated into control and experimental groups. Randomization was not relevant to the experiments using clinical cohorts.
Blinding	The IHC experiment was performed by the pathologists without any information about patient tissue. Blinding was not used for animal works and in vitro experiments (e.g. immunoblotting, immunostaining, soft agar assay, colony forming assay, cell proliferation, and flow cytometry analysis) because the same investigator was doing group allocation during data collection and/or analysis. The robust phenotype of our results is based on objective measurements.

Reporting for specific materials, systems and methods

We require information from authors about some types of materials, experimental systems and methods used in many studies. Here, indicate whether each material, system or method listed is relevant to your study. If you are not sure if a list item applies to your research, read the appropriate section before selecting a response.

Materials & experimental systems

n/a	Involved in the study
☐	☒ Antibodies
☐	☒ Eukaryotic cell lines
☒	☐ Palaeontology and archaeology
☐	☒ Animals and other organisms
☒	☐ Clinical data
☒	☐ Dual use research of concern

Methods

n/a	Involved in the study
☐	☒ ChIP-seq
☐	☒ Flow cytometry
☒	☐ MRI-based neuroimaging

Antibodies

Antibodies used

Antibodies against p53 (1:1000, Santa Cruz, sc-126), SOX2 (1:200, Santa Cruz, sc-17320), SOX10 (1:200, Santa Cruz, sc-17342), OLIG2 (1:200, R&D Systems, BAF2418), GFAP (1:200, BioLegend, 837201), β-TUBULIN III (1:200, Sigma-Aldrich, SAB4200715), NESTIN (1:200, BioLegend, 655102), Ki67 (1:200, Life Technologies, 14-5698-82), VCL (1:2000, Sigma-Aldrich, V4505), PAX6 (1:200, Biolegend, 901301), Flag (1:2000, Sigma-Aldrich, F1804), HA (1:2000, Roche, 11666606001), V5 (1:2000, Thermo Fisher Scientific, R960-25), GFP (1:2000, Santa Cruz, sc-9996), m6A (1:1000, Synaptic Systems, 202003), YTHDF2 (1:1000, Proteintech, 24744-1-AP; 1:200, Aviva, ARP67917_P050), SVIL (1:500, Sigma-Aldrich, S8695), STEM121 (1:200, Takara Bio, Y40410), human nuclear antigen (1:200, Novus Biologicals, NBP2-34342), MLL1 (1:500, Bethyl Laboratories, A300-086A), H3K4me3 (1:200, Abcam, ab8580), H3K27Ac (1:200, Abcam, ab4729), METTL3 (1:1000, Proteintech, 15073-1-AP), METTL14 (1:1000, Cell Signaling, 48699), WTAP (1:1000, ABclonal Technology, A14695), FTO (1:1000, ABclonal Technology, A3851), ALKBH5 (1:1000, ABclonal Technology, A11684), CDKN2B (1:200, Thermo Fisher Scientific, MA1-12294), and SPOCK2 (1:200, Bioss Antibodies, BS-11966R) were purchased from the indicated suppliers.

Validation

Commercial available antibodies were selected based on their antigen specificity and suggested application as described on the manufacturer's website.
Goat polyclonal anti-SOX10 Santa Cruz Biotechnology, Cat# sc-17342; https://www.scbt.com/p/sox-10-antibody-a-2
Goat polyclonal anti-SOX2 Santa Cruz Biotechnology, Cat# sc-17320; https://www.scbt.com/p/sox-2-antibody-y-17
Mouse monoclonal anti- β-TUBULIN III (12113) Sigma-Aldrich, Cat# SAB4200715; https://www.sigmaaldrich.com/US/en/product/sigma/sab4200715
Goat polyclonal anti-OLIG2 R&D Systems, Cat# BAF2418; https://www.rndsystems.com/products/human-mouse-olig2-biotinylated-antibody_baf2418
Mouse monoclonal anti-GFAP (ASTRO6) BioLegend, Cat# 837201; https://www.biolegend.com/de-de/products/anti-gfap-antibody-11057
Mouse monoclonal anti-NESTIN (Rat-401) BioLegend, Cat# 655102; https://www.biolegend.com/de-de/products/purified-anti-nestin-antibody-8496
Mouse monoclonal anti-Ki67 (SolA15) Life Technologies, Cat#14-5698-82; https://www.thermofisher.com/antibody/product/Ki-67-Antibody-clone-SolA15-Monoclonal/14-5698-82
Mouse monoclonal anti-p53 (DO-1) Santa Cruz Biotechnology, Cat# sc-126; https://www.scbt.com/p/p53-antibody-do-1?gclid=Cj0KCQiAutyfBhCMARIsAMgcRJTlpICeFERxCVnpQtnrqTBE1nNCq8nMAnkdptvrhOafx_F5mLlabSMaAgnNEALw_wcB
Mouse monoclonal anti-VCL (VIN-11-5) Sigma-Aldrich, Cat# V4505; https://www.sigmaaldrich.com/US/en/product/sigma/v4505
Rabbit polyclonal anti-YTHDF2 Proteintech, Cat# 24744-1-AP; https://www.ptglab.com/products/YTHDF2-Antibody-24744-1-AP.htm
Rabbit polyclonal anti-YTHDF2 Aviva, Cat# ARP67917_P050; https://www.avivasysbio.com/ythdf2-antibody-c-terminal-region-arp67917-p050.html
Mouse monoclonal anti-GFP (B2) Santa Cruz Biotechnology, Cat# sc-9996; https://www.scbt.com/p/gfp-antibody-b-2
Rabbit polyclonal anti-SVIL Sigma-Aldrich, Cat# S8695; https://www.sigmaaldrich.com/US/en/product/sigma/s8695
Mouse monoclonal tag anti-V5 (R960-25) Thermo Fisher Scientific, Cat# R960-25; https://www.thermofisher.com/antibody/product/V5-Tag-Antibody-Monoclonal/R960-25
Rabbit polyclonal anti-MLL1 Bethyl Laboratories, Cat# A300-086A, https://www.thermofisher.com/antibody/product/MLL1-Antibody-Polyclonal/A300-086A
Mouse monoclonal anti- nuclear antigen antibody (235-1) Novus Biologicals, Cat# NBP2-34342, https://www.novusbio.com/products/nuclear-antigen-antibody-235-1_nbp2-34342
Rabbit polyclonal anti-m6A Synaptic Systems, Cat# 202003, https://sysy.com/product/202003
Rabbit polyclonal anti-Histone H3 (tri methyl K4) antibody Abcam, Cat# ab8580, https://www.abcam.com/histone-h3-tri-methyl-k4-antibody-chip-grade-ab8580.html
Rabbit polyclonal anti-Histone H3 (acetyl K27) antibody ChIP Grade Abcam, Cat# ab4729, https://www.abcam.com/histone-h3-acetyl-k27-antibody-chip-grade-ab4729.html
Rabbit polyclonal anti-METTL3 antibody Proteintech, Cat# 15073-1-AP; https://www.ptglab.com/products/METTL3-Antibody-15073-1-AP.htm
Rabbit polyclonal anti-METTL14 Cell Signaling, Cat# 8699; https://www.cellsignal.com/products/primary-antibodies/mettl14-e2g9a-rabbit-mab/48699
Rabbit polyclonal anti-WTAP ABclonal Technology, Cat# A14695; https://abclonal.com/catalog-antibodies/WTAPRabbitpAb/A14695
Rabbit polyclonal anti-FTO ABclonal Technology, Cat# A3851; https://abclonal.com/catalog-antibodies/ELF4PolyclonalAntibody/A3851
Rabbit polyclonal anti-ALKBH5 ABclonal Technology, Cat# A11684; https://abclonal.com/catalog-antibodies/ALKBH5RabbitpAb/A11684
Mouse monoclonal anti-CDKN2B (DCS114.1) Thermo Fisher Scientific, Cat# MA1-12294; https://www.thermofisher.com/antibody/product/CDKN2B-Antibody-clone-DCS114-1-Monoclonal/MA1-12294

Rabbit polyclonal anti-SPOCK2 Bioss Antibodies, Cat# BS-11966R; https://www.biossusa.com/products/bs-11966r
Rabbit polyclonal anti PAX6 Biolegend, Cat# 901301), https://www.biolegend.com/en-us/products/purified-anti-pax-6-antibody-11511?GroupID=GROUP26
Mouse monoclonal anti-Flag (M2) Sigma-Aldrich, Cat# F1804; https://www.sigmaaldrich.com/US/en/product/sigma/f1804
Mouse monoclonal anti-HA (9E10) Roche, Cat# 11666606001; https://www.sigmaaldrich.com/US/en/product/roche/roaha

Eukaryotic cell lines

Policy information about cell lines and Sex and Gender in Research

Cell line source(s)	WT and LFS iPSCs were generated previously (PMID: 27722205).<br>H1 hESCs (WA01) were purchased from WiCell.<br>Brain tumor cell line LNZ308 was originally from ATCC.<br>HEK-293 and HEK-293T cell lines were purchased from ATCC.
Authentication	None of the cell lines used were authenticated.
Mycoplasma contamination	All cell lines tested negative for mycoplasma contamination.
Commonly misidentified lines<br>(See ICLAC register)	None of the cell lines used are listed as commonly misidentified lines in the ICLAC database.

Animals and other research organisms

Policy information about studies involving animals; ARRIVE guidelines recommended for reporting animal research, and Sex and Gender in Research

Laboratory animals	Foxn1nu mice of 8 weeks of age were purchased from The Jackson Laboratory. Mice were maintained at an ambient temperature of 70 ± 2°F and relative humidity of 30–70% under a 12-hr light/12-hr dark cycle. All animal procedures were conducted under the approval of The University of Texas Health Science Center at Houston Institutional Animal Welfare Committee.
Wild animals	No wild animals were used in this study.
Reporting on sex	n/a
Field-collected samples	No field-collected samples were used in this study.
Ethics oversight	All animal experiments were performed in accordance with The University of Texas Health Science Center at Houston Institutional Animal Welfare Committee guidelines in AAALAC-accredited barrier facility vivarium.

Note that full information on the approval of the study protocol must also be provided in the manuscript.

ChIP-seq

Data deposition

☒ Confirm that both raw and final processed data have been deposited in a public database such as GEO.

☒ Confirm that you have deposited or provided access to graph files (e.g. BED files) for the called peaks.

Data access links<br>*May remain private before publication.*	The raw and processed RNA-seq, ChIP-seq, MeRIP-seq, and eCLIP-seq data are deposited on GEO (GSE163088):<br>https://www.ncbi.nlm.nih.gov/geo/query/acc.cgi?acc=GSE163088
Files in database submission	GSM4971843 Astrocyte WT-1<br>GSM4971844 Astrocyte WT-2<br>GSM4971845 Astrocyte LFS-1<br>GSM4971846 Astrocyte LFS-2<br>GSM4971847 Astrocyte H1-1<br>GSM4971848 Astrocyte H1-2<br>GSM4971849 Astrocyte H1-3<br>GSM4971850 Astrocyte H1-245-1<br>GSM4971851 Astrocyte H1-245-2<br>GSM4971852 Astrocyte LFS-ctrl1 ov<br>GSM4971853 Astrocyte LFS-ctrl2 ov<br>GSM4971854 Astrocyte LFS-CDKN2B OV1<br>GSM4971855 Astrocyte LFS-CDKN2B OV2<br>GSM4971856 Astrocyte LFS-ctrl1 DMSO<br>GSM4971857 Astrocyte LFS-ctrl2 DMSO<br>GSM4971858 Astrocyte LFS-OICR-9429-1<br>GSM4971859 Astrocyte LFS-OICR-9429-2<br>GSM4971860 Astrocyte LFS-ctrl1<br>GSM4971861 Astrocyte LFS-YTHDF2 KD1<br>GSM4971862 Astrocyte LFS-YTHDF2 KD2

GSM4971863 Astrocyte LFS m6A input
GSM4971864 Astrocyte LFS m6A
GSM4971865 Astrocytes WT DMSO
GSM4971866 Astrocytes WT with OICR-9429
GSM4971867 Astrocytes LFS with OICR-9429
GSM4971868 Astrocytes LFS DMSO
GSM4971869 Astrocyte LFS-MLL1 KD
GSM4971870 Astrocyte WT-1 p53 CHIP
GSM4971871 Astrocyte LFS-1 p53 CHIP
GSM4971872 Astrocyte WT-1 H3K27ac CHIP
GSM4971873 Astrocyte LFS-1 H3K27ac CHIP
GSM6576357 Astrocyte LFS-1 YTHDF2 input
GSM6576358 Astrocyte LFS-1 YTHDF2 eCLIP
GSM6576359 Astrocyte LFS-2 YTHDF2 input
GSM6576360 Astrocyte LFS-2 YTHDF2 eCLIP

Genome browser session (e.g. UCSC): no longer applicable

Methodology

Replicates: All ChIP-seq experiments were performed 1 or 2 times, and confirmed with ChIP-qPCR experiments.

Sequencing depth: 150bp PE or paired-end sequencing was performed to obtain 30 million read depth

Antibodies: p53 (Santa Cruz, sc-126), m6A (Synaptic Systems, 202203), YTHDF2 (Proteintech, 24744-1-AP; Aviva, ARP67917_P050), H3K4me3 (Abcam, ab8580), and H3K27Ac (Abcam, ab4729)

Peak calling parameters: MACS2 default

Data quality:
effective genome size = 2.70e+09
band width = 300
model fold = [5, 50]
qvalue cutoff = 1.00e-02

Software:
For QC: FastQC
For alignment: Bowtie2
For ChIPseq sorting, normalization, and visualization: SAMtools, deepTools, and IGV
For peaks annotation and motifs prediction: HOMER

Flow Cytometry

Plots

Confirm that:

☒ The axis labels state the marker and fluorochrome used (e.g. CD4-FITC).

☒ The axis scales are clearly visible. Include numbers along axes only for bottom left plot of group (a 'group' is an analysis of identical markers).

☒ All plots are contour plots with outliers or pseudocolor plots.

☒ A numerical value for number of cells or percentage (with statistics) is provided.

Methodology

Sample preparation: Dissociated fresh cells were passed through a 40 µm filter to generate single cells and stained with DAPI on ice for 15 min in PBS containing 0.1% FBS according to manufacturer's instructions.

Instrument: Flow cytometry analyses were performed using a BD LSR II flow cytometer.

Software: The manufacturer's FACSDiva (BD) software was used for data collection. Flow cytometry data were analyzed using FlowJo v8.

Cell population abundance: 10,000 cells

Gating strategy: Gating live cells by DAPI staining and analyzing GFP and mCherry population. Gates drawn were based on cell populations between negative (isotype controls) and positive (single-staining) staining.

☒ Tick this box to confirm that a figure exemplifying the gating strategy is provided in the Supplementary Information.
